# Freeze–Thaw Damage of Coal Gangue–Iron Tailings Sintered Porous Bricks in Cold Region Environments

**DOI:** 10.3390/ma19091779

**Published:** 2026-04-27

**Authors:** Jing Li, Su Lu, Jiaxin Liu, Shuaihong Fan, Jianqing Tang, Shasha Li, Zhongying Li, Shunshun Ren, Zilong Liu

**Affiliations:** 1 Xinjiang College of Science & Technology, Korla 841000, China; lijingmario88@163.com; 2 College of Water Conservancy & Hydropower Engineering, Hohai University, Nanjing 210098, China; qing93@hhu.edu.cn; 3 Department of Structural Geotechnical and Building Engineering, Politecnico Di Torino, 10129 Torino, Italy; 4 College of Resources and Environmental Engineering, Inner Mongolia University of Technology, Hohhot 010051, China; fansh0515@163.com; 5 School of Qilu Transportation, Shandong University, Jinan 250002, China; lishasha0902@163.com; 6 School of Civil and Resource Engineering, University of Science and Technology Beijing, Beijing 100083, China; lzy0119@xs.ustb.edu.cn; 7 School of Civil Engineering, Qingdao University of Technology, Qingdao 266520, China; renshunshun@stu.qut.edu.cn; 8 School of Civil Engineering, Weifang University of Science and Technology, Weifang 262700, China; a85313128lzl@163.com

**Keywords:** solid waste utilization, sintered porous bricks, freeze–thaw damage, Wiener process, service life prediction

## Abstract

**Highlights:**

- Multi-scale damage mechanisms of CG-IT SPB under freeze-thaw were revealed.
- Wiener process modeled the stochastic freeze-thaw degradation.
- A method predicted frost resistance life in cold regions.
- Lab-to-nature freeze-thaw cycle equivalence was established.

**Abstract:**

Coal gangue (CG) and iron tailings (ITs) are major industrial solid wastes, and their high-value reuse is crucial for sustainable construction materials. This study explores the feasibility of fabricating sintered porous bricks using CG and ITs as primary constituents, with shale as an auxiliary component. To evaluate durability in cold regions, laboratory freeze–thaw (F-T) cycling experiments were conducted. A degradation assessment framework based on the Wiener stochastic process was developed to predict frost-resistance service life by integrating experimental data with regional climatic conditions. Results show that the fabricated bricks exhibit satisfactory initial properties, with a compressive strength of 10.6 MPa and water absorption of 13.3%. With increasing F-T cycles, compressive strength decreases significantly, accompanied by increased mass loss and water absorption. Stress–strain analysis reveals progressive stiffness reduction and a transition from brittle to ductile failure. Microstructural observations confirm degradation of the glassy phase, pore expansion, and enhanced interconnectivity. The Wiener process-based model effectively describes the stochastic accumulation of F-T damage. By establishing equivalence between laboratory and natural F-T cycles, the long-term service life of coal gangue–iron tailing sintered porous bricks (CG-IT SPBs) in cold regions is theoretically evaluated. This work provides an integrated understanding of F-T damage behavior and establishes a scientific foundation for durability-oriented design and application of such bricks in extremely cold environments.

## 1. Introduction

Industrial solid waste is an inevitable by-product of industrial development, and its large-scale generation is closely associated with the processes of industrialization and urbanization, playing a particularly important role in the construction and growth of emerging and developing economies [1,2]. However, the rapid expansion of industrial activities has also led to the severe challenge of a dramatic increase in solid waste generation [3]. If improperly managed, large quantities of industrial solid waste are disposed of through landfilling or open stockpiling, which not only occupy extensive land resources but also induce a series of environmental problems, including soil degradation, water pollution, and ecological damage. Nevertheless, given appropriate utilization strategies, these wastes may be transformed into valuable secondary resources [4,5]. Moreover, the continuous growth in resource consumption could indicate that promoting extensive reuse of significant industrial solid wastes has become an urgent requirement for alleviating the critical environmental pressure and advancing circular development [6,7].

Among different categories of industrial solid wastes, CG may represent the dominant solid residue produced throughout coal mining operations, typically accounting for about 10–20% of total raw coal output [8,9]. However, China is the world’s largest coal producer, and the accumulation of CG might have reached an extremely large scale. By 2020, the total stockpile had surpassed 7 billion tons, resulting in more than 1700 gangue dumps and occupying over ten thousand mu of land. Annual production of CG has remained high in recent years, amounting to 743 million tons in 2021, increasing to 808 million tons in 2022, and further rising to 829 million tons in 2023 [10,11,12,13,14].

Given that CG accumulates extensively, substantial occupation of land resources could indicate potential environmental risks, as spontaneous combustion may release hazardous gases such as sulfur dioxide and nitrogen oxides. Furthermore, large CG stockpiles might be associated with significant secondary pollution phenomena, such as dust dispersion and the important leachate release, thereby exerting combined adverse effects on the surrounding atmosphere, the soil, and the relevant water systems. Additionally, these impacts are compounded by engineering safety concerns, including slope instability and collapse risk [15,16,17,18]. In China, comprehensive utilization of CG has remained at a low level for an extended period; nevertheless, improvements have been achieved under circular economy policies. Therefore, statistics from 2021 may indicate the overall utilization rate of industrial solid wastes reached 56.8%, among which the utilization rate of CG exceeded 70%, while the annual growth rate of emissions was controlled within 6%, indicating progress in resource utilization of CG [19,20,21,22].

ITs are solid waste residues produced during the beneficiation of iron ore. Their output has risen over time as the steel industry has grown fast [23,24]. Data from China Mining News show that the total output of tailings in China reached 1424 billion tons in 2023. Among this amount, ITs accounted for about 617 million tons, which was 43.3% of the total. Most ITs are stored in dedicated tailings ponds. By 2024, a total of 7820 tailings ponds were under environmental supervision [25]. This large accumulation of ITs needs a large amount of land. It also requires high costs for construction and long-term operation of storage facilities. Estimates suggest that the global annual cost for the safe disposal of ITs is more than 2.2 billion USD. This cost places strong financial pressure on local governments and mining companies [26,27]. Besides economic issues, tailings ponds also create environmental risks. These risks include groundwater pollution, dust release, and land desertification. Over time, these problems may threaten nearby ecosystems and public health [28,29].

The construction sector is known as a resource- and energy-intensive industry. In recent years, its demand for wall materials has continued to rise [30,31]. Traditional clay bricks depend on large-scale soil excavation. This process causes serious damage to arable land. Because of this impact, national regulations have clearly restricted or even banned their production and use [32]. Under this situation, using bulk solid wastes such as CG and ITs to produce new wall materials is a practical option [33], This approach helps reduce the pressure from solid waste storage. It also lowers the need for natural raw materials. At the same time, it provides the construction sector with more sustainable building materials. This practice fits well with the goals of “waste-free cities” and the basic ideas of the circular economy. As a result, it brings clear environmental, social, and economic benefits.

SPBs play an important role among wall materials. This is because they show good mechanical strength, good thermal insulation, and easy construction [34,35]. In sintered brick production, industrial solid wastes are now used to partly or fully replace traditional clay. This practice has become a main route for solid waste reuse in both China and other countries [36,37,38,39,40,41,42]. To date, notable advances have been achieved in the fabrication of sintered bricks using CG, ITs, and their individual or combined applications [43]. Xu [44] fabricated high-performance sintered bricks using CG as the only raw material. When the sintering temperature was set to 1200 °C, the bricks exhibited a water absorption of 3.65% and a compressive strength of 45.61 MPa. Other studies have reported the preparation of sintered solid insulating bricks at 950 °C using CG, fly ash, and bentonite, with a CG content of 35%. During the sintering process, the combustion of residual carbon in CG promoted the transformation of aluminates and other minerals into amorphous phases, while composite silicates and mullite crystal phases were formed under thermal action, thereby imparting high strength to the bricks [45]. Yang [46] fabricated bricks from low-silicon ITs and fly ash at temperatures ranging from 900–1000 °C, confirming that ITs can substitute for traditional clay; however, the fly ash content must be carefully controlled to avoid strength deterioration. Chen [47] further optimized the mix proportions and processing parameters, proposing an appropriate mass ratio of ITs, fly ash, and clay of 84:6:10, under which bricks were successfully produced. Previous research has demonstrated that sintered bricks can be successfully prepared using ITs and CG powders as the principal raw materials, with shale and sewage sludge incorporated as auxiliary binders [42]. In general, current studies on solid waste-based sintered bricks focus on several key aspects. These aspects include raw material composition, process parameter control, basic physical and mechanical properties, and the fixation behavior of heavy metals. These studies provide basic experimental support for further promotion and practical use.

For wall materials used in cold and severely cold regions, frost resistance durability is a key performance index. It directly affects service life and structural safety [48,49]. During F-T exposure, water in the pores freezes and expands. It then thaws and shrinks. This repeated process creates high internal stress. As a result, microcracks start and grow. Surface peeling and strength loss also occur. In severe cases, structural failure may happen. In the Inner Mongolia region of China, winter periods are long and cold. Daily temperature changes are large. F-T cycles occur many times each year. Because of these conditions, wall materials must have strong frost resistance [50,51,52].

Several studies have examined the durability of solid waste-based sintered bricks. However, most studies only consider a small number of F-T cycles. Most of them also focus mainly on changes in macroscopic properties. Comprehensive monitoring of the full F-T damage evolution process, especially the accelerated deterioration stage occurring during the later service period, remains insufficiently explored. Furthermore, studies that describe degradation behavior through stochastic process–based modeling and perform long-term service life prediction are still relatively scarce [53]. In addition, most existing service life prediction models rely on deterministic approaches or empirical formulations, which are insufficient to adequately capture the randomness and cumulative nature of F-T damage, as well as the inherent uncertainty in material property degradation. Consequently, significant discrepancies may exist between predicted results and actual service life performance [54,55,56].

In view of the above considerations, this study employs CG and ITs, abundantly available in the Inner Mongolia region, as the primary raw materials, with shale introduced as an auxiliary component to adjust the chemical composition, to fabricate SPBs with a high solid waste incorporation ratio. The F-T damage evolution behavior and underlying mechanisms of the material are systematically investigated, and a stochastic process-based service life prediction model is established. The findings of this study are intended to offer theoretical guidance and practical design support for the use of CG-IT SPBs in cold-region applications. At the same time, they introduce an alternative methodological approach for evaluating durability and predicting service life of solid waste-based building materials under harsh F-T environments.

## 2. Raw Materials and Specimen Preparation

The raw materials used in this study included CG, ITs, and shale, which were collected from the Maliantan Coal Mine, Shengda Mining Co. (Beijing, China), and an abandoned shale quarry, respectively, all located in the vicinity of Tongfu Brick Factory in Ulanqab City, China. The chemical composition of each raw material is summarized in Table 1.

As indicated by the chemical composition analysis in Table 1, CG, ITs, and shale are mainly composed of SiO_2_, Al_2_O_3_, Fe_2_O_3_, CaO, and MgO, which is generally consistent with the chemical system of conventional raw materials used for sintered bricks.

**Figure 1:**
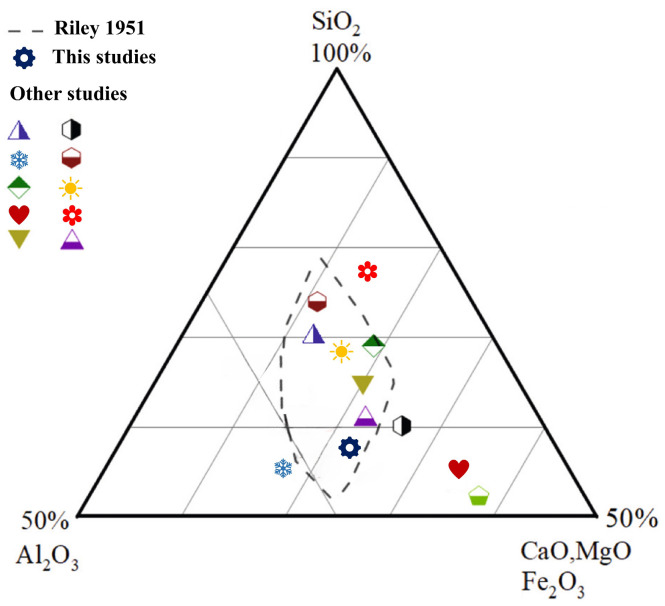
Ternary phase diagram of SiO_2_-Al_2_O_3_-Flux [57,58].

According to previous studies, the optimal chemical composition range for sintered brick raw materials is shown in Figure 1. Although the chemical compositions of the individual raw materials in this study vary, the mixture of coal gangue, iron tailings, and shale in a 4:4:2 ratio results in a composition that falls within the favorable zone for liquid phase formation. This demonstrates that this proportion can effectively promote the formation of a low-eutectic liquid phase, indicating good chemical complementarity among the three raw materials. Such a synergistic formulation suggests that they can collectively replace conventional clay in the production of sintered bricks.

**Figure 2:**
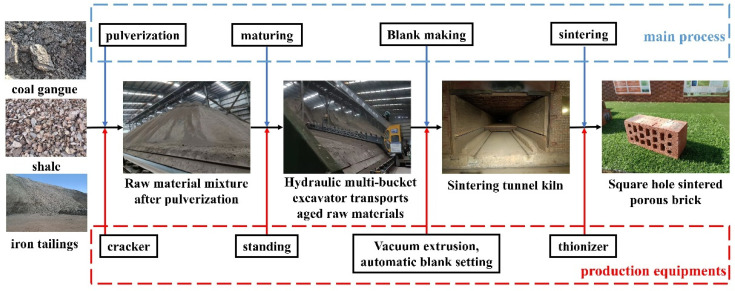
Preparation process of CG-IT SPBs.

Figure 2 shows the preparation procedure of CG-IT SPBs. In the first step, CG, ITs, and shale were blended at a mass ratio of 4:4:2 and subsequently subjected to crushing. The crushed materials were then sieved to remove oversized particles, and the retained raw materials had particle sizes controlled within the range of 0.5–2 mm [59,60]. Subsequently, molding water was added to the crushed materials to adjust the moisture content to approximately 8–9%, followed by thorough mixing and aging at room temperature for 7–10 days. After aging, the materials were remixed with additional water to achieve a moisture content of 11–13% and then transferred to a vacuum extrusion machine for forming under an extrusion pressure of 20–25 MPa. After drying, the brick bodies were sintered in a tunnel kiln at temperatures ranging from 850–1100 °C [61,62]. The appearance quality of the selected specimens complies with the relevant requirements of the Chinese standards GB/T 2542-2012 “Methods of Test for Masonry Bricks” [63] and GB/T 13544-2011 “Sintered Hollow Bricks and Hollow Blocks.” [64] The dimensions of the test specimens were 240 × 115 × 90 mm, and the pore structure consisted of square holes.

## 3. Experimental Methods

### 3.1. Mass Measurement of CG-IT SPBs Before and After F-T Cycling

Prior to testing, the specimens were dried to a constant mass in an electrically heated forced-air drying oven at 105 ± 5 °C. After cooling to room temperature, the specimens were weighed using an electronic balance with a maximum capacity of 30 kg and an accuracy of 1 g. Before the start of each test group, the initial mass of the specimen was recorded as *m* (g). After *n* F-T cycles, the mass of the specimen was recorded as *m_n_* (g). The mass loss rate of the CG-IT SPBs was determined using the following equation:

(1) $$G_{m}=\frac{m-m_{n}}{m}\times 100$$

where *G_m_* is the mass loss rate (%), *m* is the dry mass of the specimen before F-T cycling (kg), and *m_n_* is the dry mass of the specimen after F-T cycling (kg).

### 3.2. Compressive Strength Testing of CG-IT SPBs Before and After F-T Cycling

In accordance with Test Methods for Wall Bricks (GB/T 2542-2012), compressive strength test specimens were prepared using the top-and-bottom capping method (secondary molding). Prior to specimen preparation, intact brick samples were immersed in clean water at room temperature for 20–30 min, then removed and allowed to drain on a rack for 20 min. The capping mortar was prepared following the specified requirements and mixed uniformly using a mechanical mixer. The capping material complied with the requirements of Capping Mortar for Compressive Strength Testing of Wall Bricks (GB/T 25183-2010) [65].

**Figure 3:**
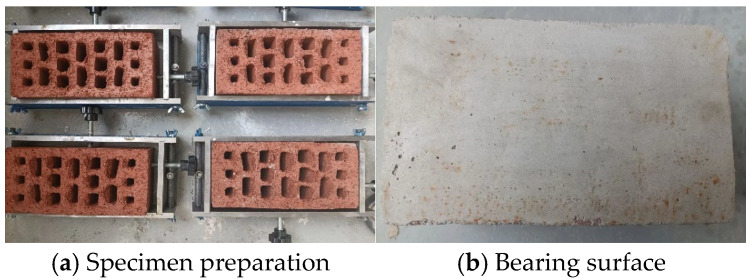
Secondary molding specimen preparation.

A release agent was applied to the inner surfaces of the molds, after which an appropriate amount of freshly mixed capping mortar was added. One load-bearing face of the undamaged specimen was placed in contact with the mortar and then positioned in the mold. The thickness of the leveling layer on the bearing surface was carefully controlled to be less than 3 mm. The mold was vibrated for 0.5–1 min and then left undisturbed until the initial setting of the mortar, after which demolding was performed. The same procedure was applied to level the opposite load-bearing surface, completing the secondary molding process (as illustrated in Figure 3).

**Figure 4:**
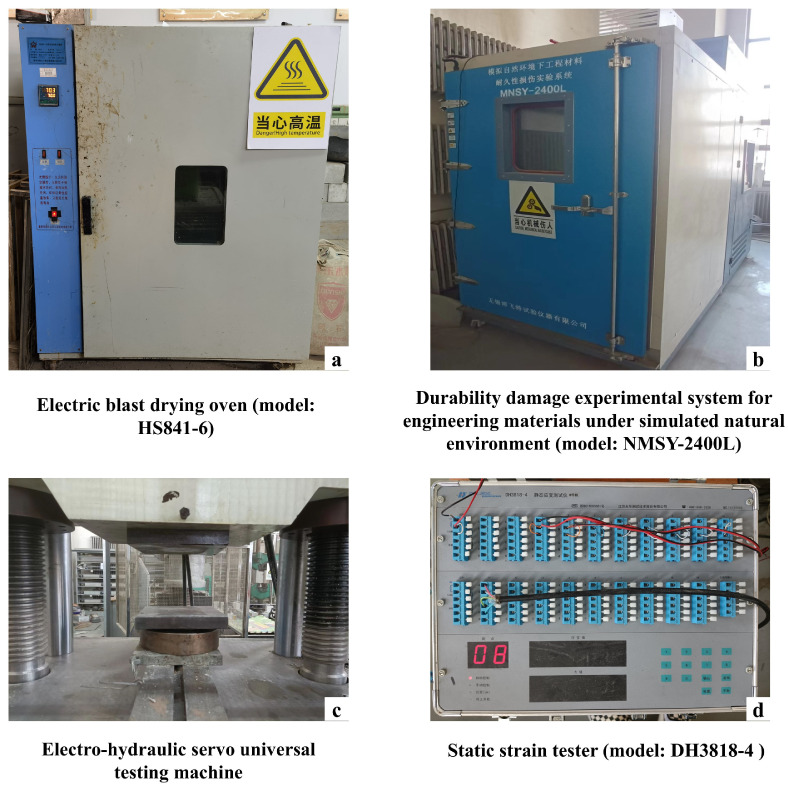
Experimental apparatus used in this study.

The compressive strength was determined with a 200 t electro-hydraulic servo universal testing system, as shown in Figure 4. During testing, the loading rate was maintained within the range of 2–6 kN/s. For each experimental condition, five specimens were tested in parallel. After each specimen failed, its maximum load was recorded and used to calculate the compressive strength. For each test group, the average value from five specimens was taken as the final compressive strength. The calculation of compressive strength followed the equation presented below:

(2) $$R_{p}=\frac{P}{L\times B}$$

where *R_p_* is the compressive strength (MPa), *P* is the maximum failure load (N), *L* is the length of the loaded surface (mm), and *B* is the width of the loaded surface (mm).

### 3.3. Water Absorption Testing of CG-IT SPBs Before and After F-T Cycling

Water absorption was measured following the procedure specified in GB/T 2542-2012. Before testing, the specimens were dried in a forced-air oven until a constant mass was reached. During the drying process, the mass difference between two consecutive weighings did not exceed 0.2%, with a time interval of 2 h between measurements. After the drying process, the mass of the specimen was measured and denoted as *m*_0_.

After the preceding procedure, the specimens were submerged in water maintained at 10–30 °C for a duration of 24 h. After the immersion step, the specimens were removed from the liquid and excess water on the surface was gently wiped off using a moist cloth. The specimens were then promptly weighed to determine the wet mass *m*_24_. For each test condition, five specimens were measured, and the arithmetic mean was adopted as the final result. Water absorption was determined using the equation presented below:

(3) $$W_{24}=\frac{m_{24}-m_{0}}{m_{0}}\times 100$$

where *W*_24_ is the 24 h water absorption of the specimen at room temperature (%), *m*_0_ is the dry mass of the specimen (kg), and *m*_24_ is the wet mass of the specimen after 24 h of water immersion (kg).

### 3.4. Bulk Density Testing of CG-IT SPBs Before and After F-T Cycling

The bulk density of the specimens was measured following GB/T 2542-2012. Before testing, the samples were placed in a forced-air oven and dried until a constant mass was reached. During the drying process, the mass difference between two consecutive weighings did not exceed 0.2%, with a time interval of 2 h between measurements. After drying, the dry mass of each specimen was measured, and the specimen dimensions were determined.

The length and width of each specimen were measured at the midpoint of the two large faces, and the height was measured at the midpoint of the two beds. If defects or protrusions were present at the measuring locations, measurements were taken adjacent to these areas, selecting the most unfavorable side. The bulk density was calculated using the following equation:

(4) $$\rho =\frac{m}{V}\times 10^{9}$$

where *ρ* is the bulk density (kg/m^3^), *m* is the dry mass of the specimen (kg), and *V* is the specimen volume (mm^3^).

### 3.5. F-T Cycling Test of CG-IT SPBs

The F-T cycling test was carried out in accordance with GB/T 2542-2012. Before the test, each specimen was brushed to remove any loose particles and surface contaminants. Drying was carried out in a forced-air oven until a constant mass was achieved, defined as a mass variation of no more than 0.2% between two successive measurements taken at 2 h intervals. Upon completion of drying, the specimens were visually examined, and surface defects such as corner loss and cracking were identified and recorded.

The pretreated specimens were immersed in water at 10–20 °C for 24 h, removed, and wiped with a damp cloth to eliminate surface moisture. Subsequently, the specimens were placed vertically on their large faces with a spacing greater than 20 mm in a freezing chamber that had been pre-cooled to below −15 °C. Timing commenced when the chamber temperature reached −15 °C. Each F-T cycle consisted of freezing the specimens at −15 to −20 °C for a period of 3 h, followed by thawing through immersion in water maintained at 10–20 °C for 2 h.

The numbers of F-T cycles were selected as 0, 10, 20, 30, 40, 50, 60, and 70. After each interval of 10 cycles, the specimen mass, water absorption, compressive strength, and bulk density were tested, and the surface condition was visually documented. The drying process was carried out using an HS841-6 electric forced-air drying oven manufactured by Shanghai Shouli Industrial Co., Ltd., Shanghai, China. The F-T tests were performed using an NMSY-2400L durability damage testing system for engineering materials under simulated natural environmental conditions, manufactured by Wuxi Bofeite Testing Instrument Co., Ltd., Wuxi, China, as shown in Figure 4.

To examine the stress–strain behavior of CG-IT SPBs both before and after F-T cycling, axial compression tests were performed, and the corresponding stress–strain curves of the specimens were obtained. Lead wires were connected to a DH3818-4 static strain testing instrument (Figure 4) using a half-bridge wiring configuration to measure specimen strain.

Strain gauges were attached at the midline positions on two opposite faces of each specimen. Prior to measurement, the strain gauges and lead wires were labeled to ensure correspondence with the test data. Due to surface unevenness, the specimen surfaces were polished before strain gauge installation.

The dimensions of the loaded surfaces were measured using a vernier caliper, with three measurements taken and averaged as the final value. The specimens were placed evenly and level on the loading platen, and the load was applied perpendicular to the loaded surface in a uniform manner to avoid the influence of impact and vibration. Loading was continued until complete failure of the specimen, and the maximum load at failure was recorded. The loading rate was controlled within 0.1–1 kN/s.

## 4. Results and Discussion

### 4.1. Macroscopic Morphological Changes of CG-IT SPBs Before and After F-T Cycling

**Figure 5:**
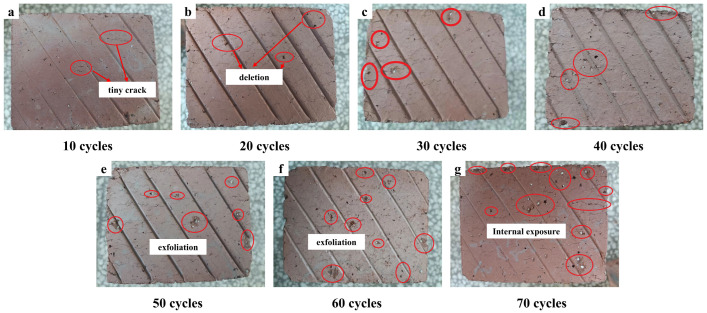
Macroscopic appearance changes on the side surfaces of specimens under F-T cycling. (**a**) 10 cycles, showing tiny cracks; (**b**) 20 cycles, showing defect initiation;(**c**) 30 cycles, showing development of surface defects; (**d**) 40 cycles, showing increased damage; (**e**) 50 cycles, showing surface exfoliation; (**f**) 60 cycles, showing intensified exfoliation; (**g**) 70 cycles, showing internal exposure.

Figure 5 illustrates the macroscopic morphological changes on the side surfaces of CG-IT SPBs subjected to different numbers of F-T cycles. As illustrated in the figure, surface damage features became increasingly pronounced as the number of F-T cycles increased. After 10–20 F-T cycles (Figure 5a,b), fine microcracks began to appear on the surface, accompanied by slight localized bulging, while the surrounding edges and corners remained intact without noticeable loss. When the number of F-T cycles reached 30 (Figure 5c), the number of surface cracks increased markedly, and a small number of approximately circular local spalling defects were observed, with white substances visible in the damaged areas.

As the F-T cycles further increased to 40–60 (Figure 5d–f), the surface cracks continued to propagate and widen, and both the number and extent of spalled regions increased. The accumulation of white deposits intensified, while localized spalling developed at the edges and corners, giving rise to a generally roughened and uneven surface morphology. After 70 F-T cycles (Figure 5g), the damaged regions expanded significantly, with greater exposure of the internal material, and severe deterioration was observed, particularly at the edges and corners.

It is noteworthy that throughout the entire F-T testing process, only a limited number of microcracks formed on the inner walls of the pores of the SPBs, and no obvious spalling or material loss was observed. This phenomenon can be explained by the comparatively smooth surfaces of the pore walls and the smaller amount of initial flaws formed during specimen fabrication by vacuum extrusion. Moreover, the mechanical properties of this region were further enhanced during the sintering process, thereby imparting superior resistance to F-T damage compared with other parts of the brick.

The locally spalled regions highlighted in Figure 5 may be influenced not only by freeze–thaw damage but also by the particle size distribution of the raw materials. In this study, the raw materials were crushed and sieved to control the particle size within 0.5–2 mm. Nevertheless, a small number of oversized or irregular particles may exist, which could create stress concentration points or, during sintering, generate thermal expansion mismatch with the matrix, thus forming initial weak interfaces. However, with increasing freeze–thaw cycles, the damage progressively intensifies, indicating that freeze–thaw action is the dominant factor in damage evolution, while the effect of particle size serves as a secondary triggering factor [66,67]. To minimize this influence in future production, particle size could be further controlled below 1 mm, and mixing homogeneity could be enhanced.

### 4.2. Mass Variation of CG-IT SPBs Before and After F-T Cycling

**Figure 6:**
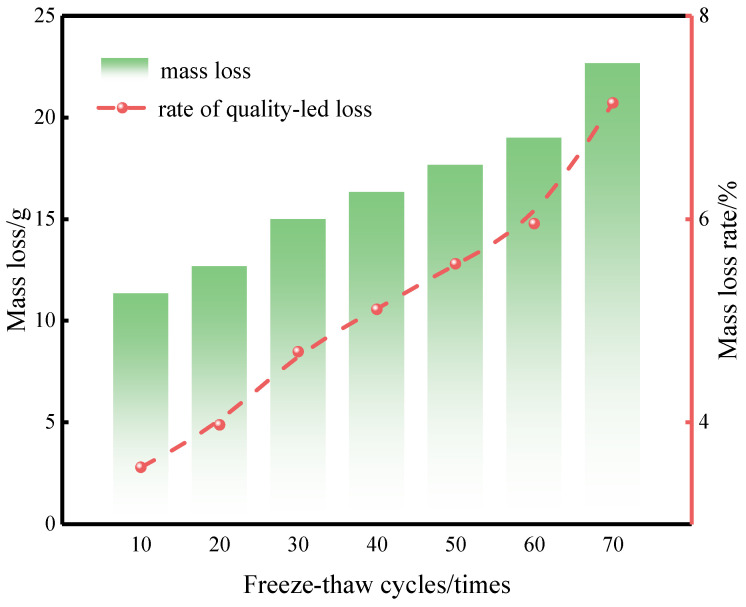
Mass loss of CG-IT SPBs under different numbers of F-T cycles.

Figure 6 illustrates how the mass of CG-IT SPBs changes with increasing numbers of F-T cycles. As indicated in the figure, specimen mass loss increased steadily as the number of F-T cycles rose, reaching a cumulative loss of about 22.7 g. During the initial stage of 10–20 F-T cycles, the increase in mass loss was relatively gradual. After 30 cycles, however, the mass loss intensified noticeably, with the mass loss rate increasing by approximately 0.72%. This behavior is mainly attributed to the onset of localized surface spalling.

Within the range of 30–60 F-T cycles, the mass loss continued to increase at a relatively steady rate, and the incremental mass loss rate at each stage remained below 0.5%. In contrast, after the number of F-T cycles increased to 70, the mass loss increased clearly. The loss rate rose by about 1.19%. This sharp rise occurred because the specimens entered a stage of faster damage under long-term F-T action. At this stage, surface spalling became more serious. Damage also appeared at the edges and corners. As a result, the mass dropped strongly. This result shows that the structural integrity of the bricks degraded quickly during the later stage of F-T cycling.

### 4.3. Variation in Compressive Strength of CG-IT SPBs Before and After F-T Cycling

**Figure 7:**
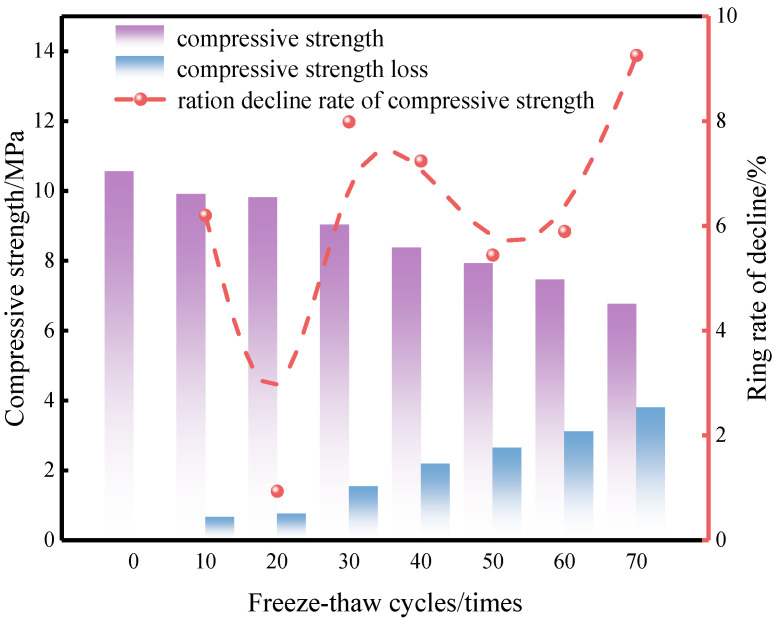
Compressive strength loss of CG-IT SPBs under different numbers of F-T cycles.

The evolution of CG-IT SPB compressive strength under different F-T cycle numbers is displayed in Figure 7. As shown in the figure, the compressive strength of the specimens exhibited a gradual declining trend with increasing F-T cycles. Before F-T cycling, the specimens exhibited an initial compressive strength of 10.6 MPa. The strength dropped by nearly 6.2% following 10 F-T cycles, which can be mainly attributed to the initiation of internal microdefects and microcracks induced by early-stage F-T action. At 20 cycles, the rate of strength reduction slowed, with a decrease of only 0.9%.

Within the range of 30–60 F-T cycles, the compressive strength continued to decline in a relatively uniform manner, with a reduction of approximately 5–8% at each stage, indicating a stable degradation trend. After 70 F-T cycles, the compressive strength decreased to 6.8 MPa, representing an overall loss of approximately 35.8%.

The decrease in strength is mainly caused by internal stress from the water–ice phase change during F-T cycling. Under saturated conditions, water gradually enters the pores of the brick. It also moves into existing microcracks. Inside these spaces, the water freezes and thaws again and again. Because the solid matrix limits volume change, ice expansion presses on the pore walls. This process leads to a slow increase in internal pore pressure. At the early stage of F-T exposure, the material can still resist these stresses. As the number of cycles increases, damage keeps building up and the material weakens step by step. When the pore-induced stress becomes higher than the load-bearing capacity, pore structure damage occurs. Microcracks then grow and link together. In the end, the compressive strength drops clearly [68].

### 4.4. Variation in Water Absorption of CG-IT SPBs Before and After F-T Cycling

**Figure 8:**
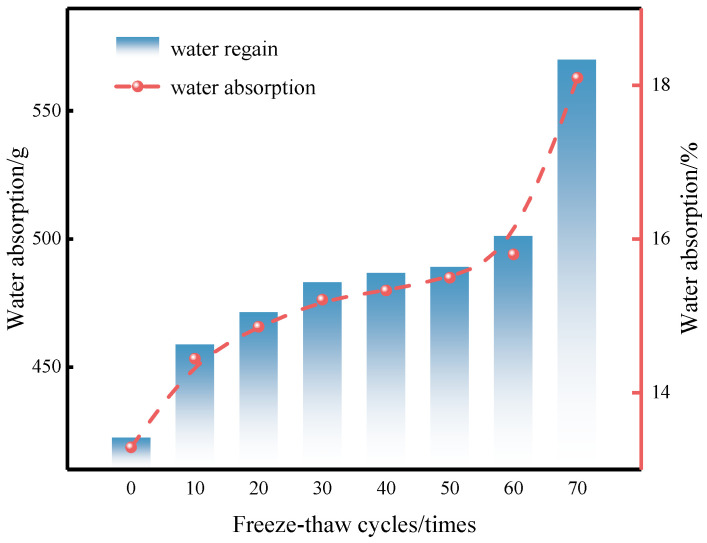
Variation in water absorption of CG-IT SPBs under different numbers of F-T cycles.

Water absorption is an important indicator reflecting the internal pore structure of materials, and its variation can directly characterize pore evolution during F-T processes [69]. Figure 8 shows how the water absorption of CG-IT SPBs varies with the number of F-T cycles. The results indicate that water absorption increases steadily as the F-T cycles progress.

At the early stage of F-T cycling (10 cycles), the water absorption increased by approximately 1.1% compared with the initial value. Up to 60 F-T cycles, the water absorption increased slowly and followed an almost linear trend. This result shows that the pore structure stayed in a relatively stable stage during this period. After 70 cycles, a clear change appeared. The water absorption increased sharply by about 3%. The absorbed water mass reached about 570 g. This sudden rise mainly comes from stronger internal damage in the later stage of F-T cycling. At this stage, the number of open pores increased greatly. The connections between pores also became stronger. As a result, the water transport pathways and storage capacity of the material were significantly improved. This macroscopic behavior is in good agreement with the pore enlargement and interconnection features revealed by microscopic morphological observations.

### 4.5. Variation in Bulk Density of CG-IT SPBs Before and After F-T Cycling

**Figure 9:**
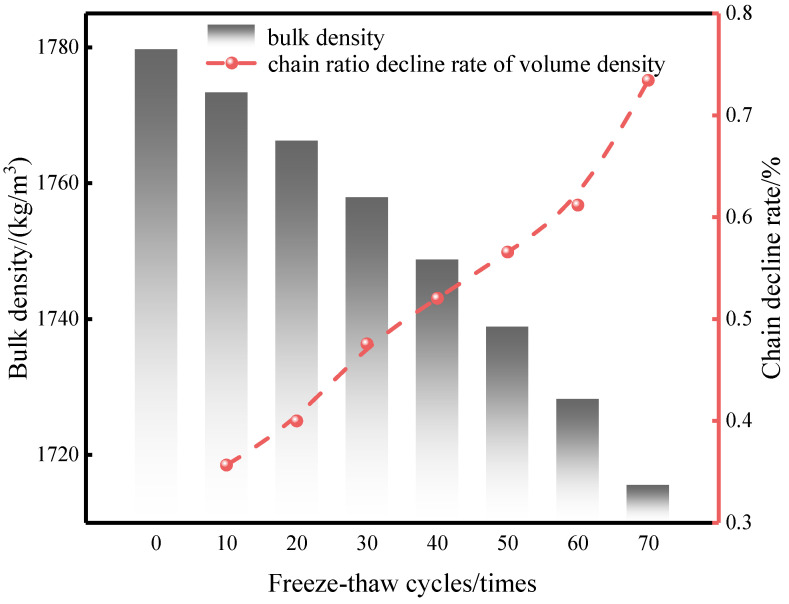
Variation in bulk density of CG-IT SPBs under different numbers of F-T cycles.

Bulk density is closely related to the pore structure and overall compactness of a specimen, and its variation can indirectly reflect mass loss and volumetric deformation induced by F-T action [70]. Figure 9 presents the relationship between bulk density and the number of F-T cycles. The results show that the bulk density decreases gradually as the number of F-T cycles increases; however, the magnitude of reduction was relatively limited, with a total decrease of less than 1%.

During F-T cycling, the specimen mass gradually decreased due to surface spalling and damage at edges and corners. However, because no significant changes occurred in the geometric dimensions of the bricks in the dried state, the resulting reduction in bulk density remained small. This indicates that within the F-T range considered in this study, the CG-IT SPBs maintained good structural integrity and high volumetric stability, with no pronounced macroscopic expansion or shrinkage induced by F-T action. The slight decrease in bulk density is therefore mainly attributed to mass loss rather than significant volumetric change, further suggesting that F-T damage is primarily manifested as surface erosion and pore development, without causing overall structural collapse or substantial deformation.

### 4.6. Evolution of Damage Degree of CG-IT SPBs After F-T Cycling

To quantitatively characterize the performance degradation of CG-IT SPBs under F-T cycling, damage mechanics theory was adopted. Mass, compressive strength, water absorption, and bulk density were selected as the primary indicators for performance evaluation [48]. The damage degree associated with each parameter was determined according to Equation (5), enabling a systematic assessment of the structural damage state of the material at various stages of F-T cycling.

(5) $$D_{\alpha }=\frac{\alpha_{0}-\alpha_{n}}{\alpha_{0}}$$

*D_α_* represents the damage degree of the CG-IT SPBs with respect to indicator *α*; *α*_0_ denotes the value of the corresponding indicator before F-T cycling; *α_n_* denotes the value of the indicator after F-T cycling. This formulation reflects, in a normalized manner, the degradation ratio of each performance parameter relative to its initial state, and provides an intuitive description of the cumulative evolution of F-T damage.

**Figure 10:**
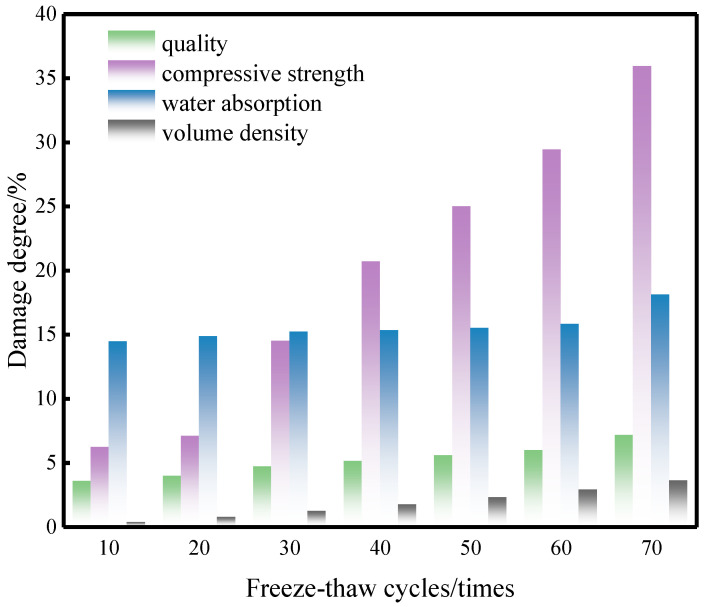
Variation in damage degrees of different indicators of CG-IT SPBs after F-T cycling.

Figure 10 presents the evolution of damage degrees for different evaluation indicators as a function of F-T cycles. In general, all damage degree curves show a progressive increasing tendency with increasing cycle numbers, indicating that the material experiences continuous and irreversible performance deterioration under F-T conditions. Clear differences can be observed in the sensitivity of various indicators to F-T action. After 70 cycles, the damage degrees corresponding to mass, compressive strength, water absorption, and bulk density reached 7.1%, 36%, 18%, and 3.6%, respectively.

Among these parameters, compressive strength exhibits the highest damage degree, suggesting that mechanical properties are most susceptible to F-T degradation. By contrast, bulk density shows the lowest damage degree, implying that the macroscopic geometric dimensions of the specimens remain relatively stable during cycling and that mass loss and pore structure evolution exert only a limited influence on bulk density. These observations further indicate that F-T damage in CG-IT SPBs is predominantly associated with internal microstructural deterioration and mechanical performance degradation, while apparent volumetric changes are relatively insignificant.

### 4.7. Stress-Strain Relationship of CG-IT SPBs After F-T Cycling

**Figure 11:**
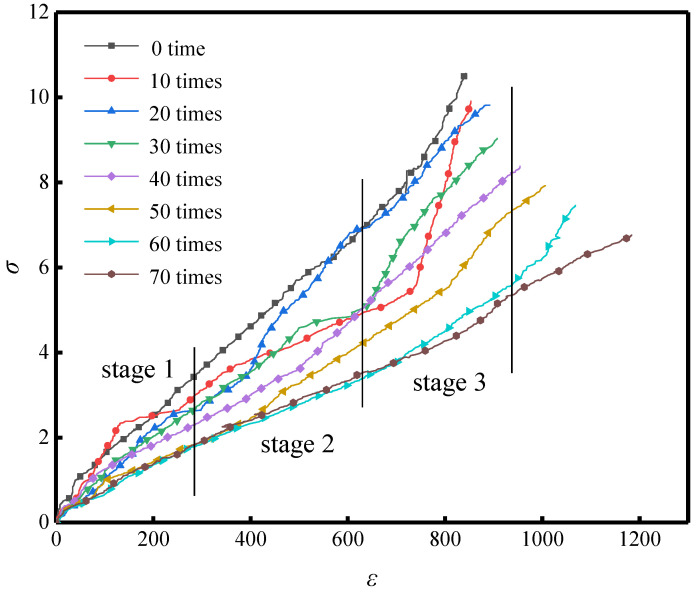
Stress–strain curves of CG-IT SPBs under different numbers of F-T cycles.

The macroscopic performance of materials is closely associated with their microstructural characteristics, and their degradation behavior directly affects structural mechanical responses. In this study, uniaxial compression tests were conducted to obtain the stress (*σ*)–strain (*ε*) data of CG-IT SPBs subjected to different numbers of F-T cycles. The corresponding stress–strain curves were fitted using Origin software v9.1, as shown in Figure 11.

The results indicate that, although the specimens experienced different numbers of F-T cycles, their stress–strain curves maintain similar overall profiles. This consistency suggests that a constitutive relationship can be reasonably defined for the material under varying F-T conditions. With an increasing number of cycles, the peak stress decreases progressively, indicating an ongoing loss of load-carrying capacity. In addition, at the same stress level, the corresponding strain becomes markedly larger, demonstrating stiffness degradation caused by F-T action and an associated decrease in elastic modulus [71].

Based on the observed crack growth and failure features during testing, the full stress–strain response of CG-IT SPBs under uniaxial compression can be divided into three stages:

Stage I: When the load reaches about 30–40% of the peak load, the stress–strain curve is almost linear. At a constant loading rate, the deformation is mainly elastic. Most deformation can recover after unloading. No clear plastic deformation appears. No visible cracks are found. Stage II: As the applied load increases to approximately 40–80% of the peak level, small surface cracks start to form, and the strain rises at a faster rate with further loading. The stress–strain curve starts to deviate from a straight line. The material changes from elastic to elasto-plastic behavior. Internal damage also starts to build up. Stage III: As the load continues to increase toward the peak stress, the stored elastic energy becomes higher than the energy needed for crack growth. The material then enters a stage of rapid crack development. Surface cracks increase and extend quickly. Many short and dense cracks form near the loading platens. Internal crushing sounds are often heard. As the ultimate load is approached, cracks gradually link into groups. This process causes serious internal damage. The effective load-transfer paths keep decreasing. In the end, longitudinal splitting and spalling failure occur. During this stage, the stress–strain curve shows a steadily decreasing slope. Strong nonlinearity and plastic deformation are also observed until the load-bearing capacity is fully lost.

### 4.8. Microscopic Morphological Analysis of F-T Damage in CG-IT SPBs

**Figure 12:**
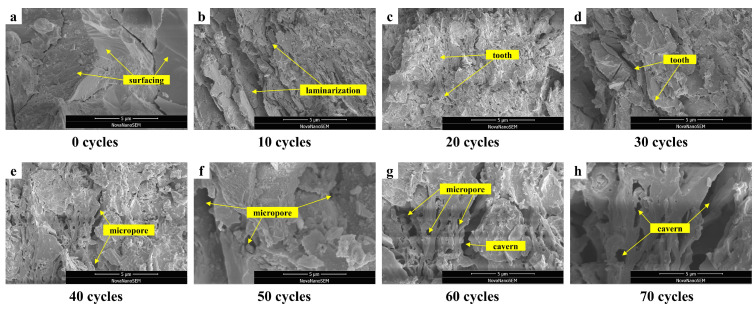
Microstructural damage evolution of CG-IT SPBs under different numbers of F-T cycles. (**a**) 0 cycles, showing a relatively intact surface; (**b**) 10 cycles, showing laminarization; (**c**) 20 cycles, showing the formation of tooth-like structures; (**d**) 30 cycles, showing further development of tooth-like features; (**e**) 40 cycles, showing the appearance of micropores; (**f**) 50 cycles, showing an increase in microporosity; (**g**) 60 cycles, showing the coexistence of micropores and caverns; (**h**) 70 cycles, showing well-developed caverns.

To better explain the damage processes of CG-IT SPBs during F-T exposure, scanning electron microscopy (SEM) was employed to analyze the microstructure of specimens after different numbers of F-T cycles. Combined with the results of macroscopic performance tests, the damage evolution process was systematically interpreted. As shown in Figure 12a, the specimen without F-T exposure presents a relatively smooth surface and a continuous lamellar microstructure, with only a limited number of pre-existing microcracks. The microstructure appears dense overall, which is consistent with the high initial compressive strength and the low water absorption observed at the macroscopic scale.

After 10–20 F-T cycles (Figure 12b,c), the specimen surface becomes noticeably rougher, and localized spalling of the lamellar structure is observed, accompanied by the emergence of microvoids. At this stage, macroscopic tests reveal the initiation of cumulative mass loss and a slight decrease in compressive strength, indicating that F-T damage is no longer confined to isolated defects but has begun to develop on the material surface.

When the F-T cycles increase to 30–40 (Figure 12d,e), the surface becomes rougher. Repeated F-T action clearly weakens the bonding between the glassy phase and the matrix [50], because of this change, crystalline phases become more exposed. Frost heave-related damage also starts to develop. As a result, microvoids grow and slowly connect with each other. At the macro scale, this microstructural change matches a clear increase in water absorption. The more developed pore network makes water transport and storage easier. At the same time, the compressive strength keeps decreasing. This trend shows the continued weakening of internal structural bonding.

At 50–60 F-T cycles (Figure 12f,g), surface spalling becomes more obvious. The pore shape turns highly irregular. Some pores show a “narrow-mouth, wide-body” form. This shape limits the release of meltwater during thawing. It also causes local strain concentration. Because of this effect, damage around the pore walls becomes more severe. At the same time, strong salt crystallization appears at the micro scale. This process is caused by the movement of soluble salts toward the surface with moisture flow. This feature agrees well with the efflorescence observed during the tests [72]. At this stage, the specimens enter a phase of faster deterioration. This stage shows a clear rise in mass loss rate. It also shows a sharp drop in compressive strength and a further increase in water absorption.

After 70 F-T cycles (Figure 12h), the glassy phase is mostly removed. The crystalline phases become loose. Many large pores form across the microstructure. These features indicate serious damage to material integrity. This microstructural change matches the strong decline in macroscopic mechanical behavior. The compressive strength drops by about 35.8%. Water absorption also increases greatly. These results confirm that F-T action causes irreversible structural damage. They also strongly support the damage trends identified earlier using mass, compressive strength, and water absorption as evaluation indicators.

During the sintering of CG-IT SPBs, SiO_2_, Al_2_O_3_, and fluxing agents in the raw materials undergo solid-state reactions and liquid-phase sintering at high temperatures, forming crystalline phases primarily composed of mullite, quartz, and sodium feldspar. From a thermodynamic perspective, components with lower thermal stability gradually decompose during sintering, and their decomposition products participate in the formation of new crystalline phases. These newly formed phases exhibit higher thermodynamic stability and, upon cooling, provide rigidity and structural stability, while the glass phase serves as a binder and filler, collectively forming the microstructural framework of the bricks. Additionally, the glassy melt formed at high temperatures penetrates along grain boundaries and pores, promoting densification of the brick body [42,62,73].

During F-T cycles, the stable crystalline phases effectively resist the destructive stresses generated by ice crystal expansion, suppressing the initiation and propagation of microcracks [74]. This behavior is consistent with the progressive reduction in compressive strength and microstructural damage observed in this study after F-T exposure. Specifically, the glass phase, having relatively lower thermodynamic stability, degrades first, while the crystalline framework remains largely intact, thereby delaying the overall failure of the material.

## 5. F-T Resistance Life Prediction of CG-IT SPBs Based on the Wiener Process

The Wiener process is a classic stochastic model. It was first used to describe Brownian motion. It represents the random paths of particles under many small and independent interactions. This model has been widely used in physics and engineering. It is often applied to describe the random behavior of particles in fluids or gases [75]. The Wiener process has a clear theoretical basis. It is also easy to analyze. Because of these features, it is suitable for describing performance degradation caused by the gradual buildup of random micro-scale damage.

During F-T cycling, water inside the brick pores freezes and melts again and again. Each phase change causes random damage to the material. This process leads to a gradual loss of performance. As the number of F-T cycles increases, damage from the water–ice change keeps accumulating at the micro level. This accumulation finally appears as continuous degradation of macroscopic properties. This degradation process shows both randomness and accumulation. Because of these characteristics, the Wiener process provides a solid theoretical basis for modeling and analyzing the F-T damage evolution of CG-IT SPBs.

### 5.1. Model Development

The general form of the Wiener process can be expressed as follows:

(6) $$B\left(t\right)=\gamma t+\eta W\left(t\right)$$

where *γ* is the drift coefficient, which primarily characterizes the degradation rate; *η* is the diffusion coefficient of the process; *t* denotes time; *B*(*t*) represents the degradation amount of CG-IT SPBs during F-T erosion; and *W*(*t*) is a standard Wiener process.

The service life of a specimen can be defined as the time at which its performance degradation first reaches a specified failure threshold. To accurately predict the service life of the specimens subjected to F-T cycling, the relevant provisions of GB/T 2542-2012 were adopted, and the relative mass loss was selected as the degradation indicator and failure criterion. Assuming that the failure threshold of CG-IT SPBs is *P* (*P* > 0), the specimen lifetime *T* satisfies the following:

(7) $$T=inf\left\{t|B\left(t\right)>P,t\geq 0\right\}$$

Let *f*(*x_k_*,*t*) denote the probability density function of *B*(*t*) at time *t*. Then, the probability that the CG-IT SPBs remain in normal service within time t can be expressed as

(8) $$P\{T>t\}=P\{B(t)<P\}=\int_{-\infty }^{P}f\left(x_{k},t\right)dx$$

It can be seen that once the probability density function *f*(*x_k_*,*t*) is determined, the distribution of the lifetime *T* can be obtained. By applying the Fokker–Planck (F-P) equation [76], the corresponding probability density function can be derived as follows:

(9) $$f\left(x,t\right)=\frac{1}{\eta \sqrt{2\pi t}}\left\{exp\left[-\frac{{\left(x-\gamma t\right)}^{2}}{2\eta^{2}t}\right]-exp\left(\frac{2\gamma P}{\eta^{2}}\right)exp\left[-\frac{\left(x-2P-\gamma t\right)}{2\eta^{2}t}\right]\right\}$$

By substituting Equation (9) into Equation (8), the distribution function can be obtained as follows:

(10) $$F_{T}\left(t\right)=\Phi \left(\frac{\gamma t-P}{\eta \sqrt{t}}\right)+exp\left(\frac{2\gamma P}{\eta^{2}}\right)\Phi \left(\frac{-P-\gamma t}{\eta \sqrt{t}}\right)$$

By analytical derivation, the probability density function together with the reliability function corresponding to the instant at which the degradation index reaches the failure threshold can be expressed as follows:

(11) $$D\left(t\right)=1-F_{T}(t)=\phi \left(\frac{P-\gamma t}{\eta \sqrt{t}}\right)-exp\left(\frac{2\gamma P}{\eta^{2}}\right)\phi \left(\frac{-P-\gamma t}{\eta \sqrt{t}}\right)$$

(12) $$f\left(t\right)=\frac{p}{\sqrt{2\pi \eta^{2}t^{3}}}\exp\left[-\frac{{\left(P-\gamma t\right)}^{2}}{2\eta^{2}t}\right]$$

where *ϕ* denotes the standard normal distribution function.

### 5.2. Model Validation

Prior to calculation, the experimental data must be examined to verify whether they satisfy the properties of a Wiener process. A one-dimensional Wiener process with drift should meet the following conditions [77]:

1. Δ*B* = *B*(*t* + Δ*t*) − *B*(*t*)~*N*(*m*Δ*t*, *b*^2^Δ*t*)
2. *B*(*t*) has independent increments; that is, for any 0 ≤ *t*_0_ < *t*_1_ < ⋯< *t_n_*, *n* ∈ *N*^+^, the increments *B*(*t*_1_) − *B*(*t*_0_), *B*(*t*_2_) − *B*(*t*_1_), ⋯, *B*(*t_n_*) − *B*(*t_n−_*_1_) are mutually independent;
3. *B*(0) = 0.

**Figure 13:**
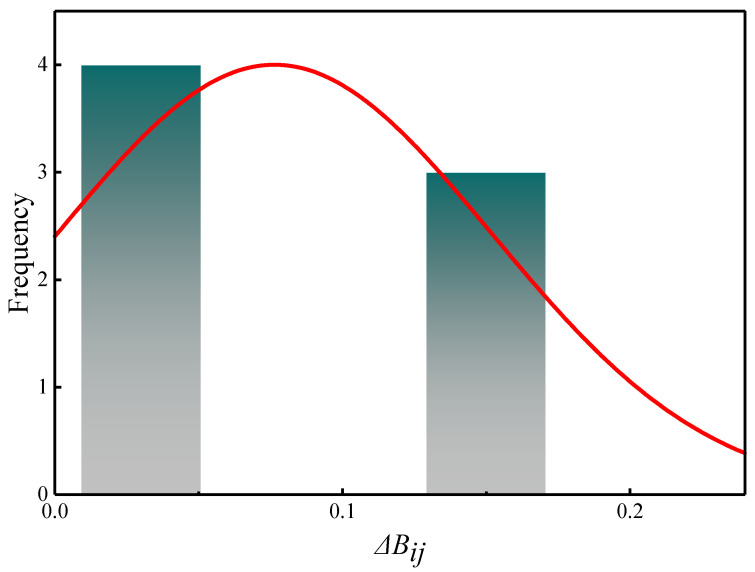
Statistical histogram of relative mass loss.

Based on the experimentally measured relative mass loss data, a statistical histogram was employed to examine the data distribution, and the results are shown in Figure 13. The analysis indicates that the relative mass loss approximately follows a normal distribution. According to statistical theory, if a degradation indicator follows a normal distribution and its evolution process exhibits continuous and independent increments, the degradation process can be regarded as a one-dimensional continuous-time stochastic process. Since the relative mass loss of CG-IT SPBs under F-T cycling satisfies the above conditions, the frost-resistance degradation behavior can be considered to conform to the assumptions of a one-dimensional Wiener process with drift. Furthermore, the Kolmogorov–Smirnov test yielded a statistic of D = 0.0875 (*p* > 0.05), indicating that the normality assumption is valid and further supporting the applicability of the Wiener process.

### 5.3. Parameter Calculation

By integrating Equations (11) and (12), the likelihood function of the Wiener process can be derived as follows:

(13) $$L\left(\gamma ,\eta^{2}\right)=\prod_{i=1}^{a}\prod_{j=1}^{b}\frac{1}{\sqrt{2\eta^{2}\pi \Delta t_{ij}}}exp\left[-\frac{\left(\Delta B_{ij}-\gamma t_{ij}\right)}{2\eta^{2}\Delta t_{ij}}\right]$$

where Δ*B_ij_* denotes the degradation increment of the j-th specimen over the time interval Δ*t_ij_*.

By applying the maximum likelihood estimation (MLE) method [78], the functional expressions of the parameters *γ* and *η* can be obtained as follows:

(14) $$\gamma =\frac{\sum_{i=1}^{a}B_{ib}}{\sum_{i=1}^{a}t_{ib}}$$

(15) $$\eta^{2}=\frac{1}{\sum_{i=1}^{a}b}\left[\sum_{i=1}^{a}\sum_{j=1}^{b}\frac{{\left(\Delta B_{ij}\right)}^{2}}{\Delta t_{ij}}-\frac{{\left(\sum_{i=1}^{a}B_{ib}\right)}^{2}}{\sum_{i=1}^{a}t_{ib}}\right]$$

Based on Equations (14) and (15), the estimated parameters are obtained as *γ* = 0.0000819 and *η*^2^ = 0.000185.

**Figure 14:**
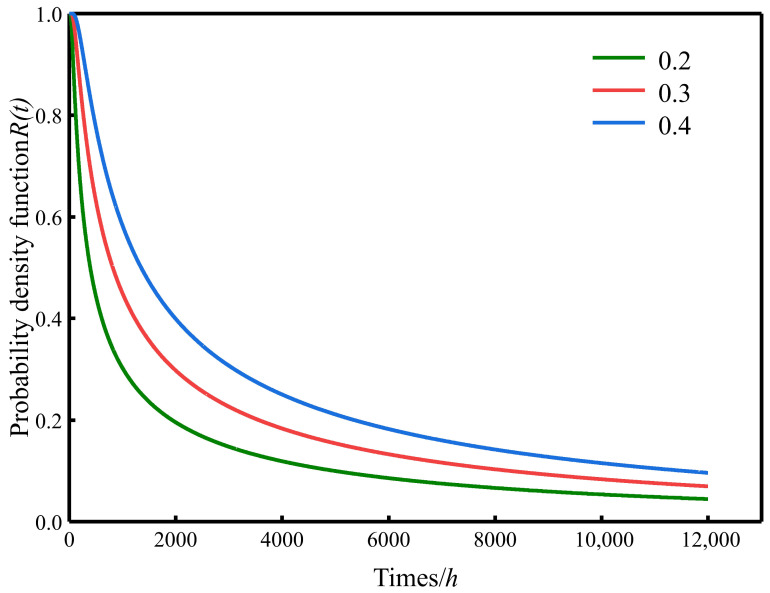
Reliability function curve of the specimen.

**Figure 15:**
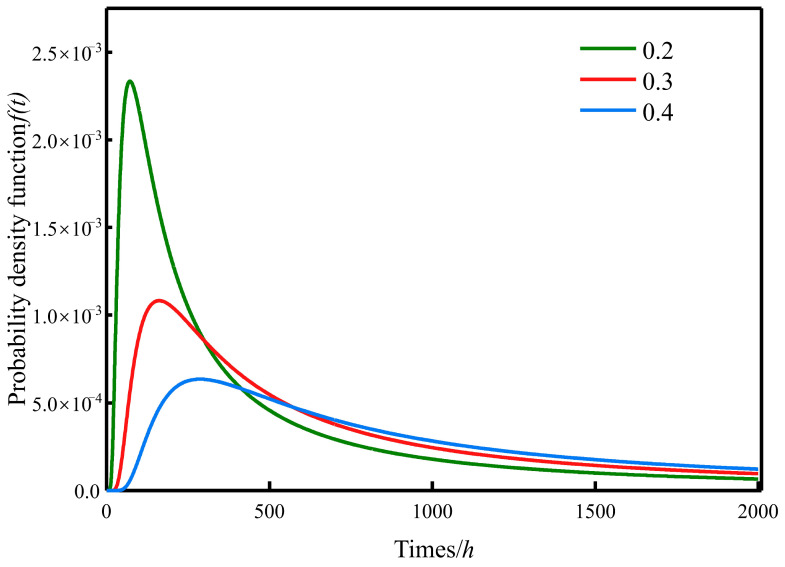
Probability density function curve of the specimen.

By substituting these parameters into Equations (11) and (12), the probability density function and reliability function of the specimens are derived. The corresponding curves were fitted using Origin software, and the results are shown in Figure 14 and Figure 15.

Analysis of the functional curves in Figure 14 indicates that, as the failure threshold decreases, the overall curve exhibits a rapid downward trend. When the threshold is reduced to a certain range, however, the rate of decline decreases markedly and the curve remains relatively stable over an extended period. This behavior suggests that the frost resistance of CG-IT SPBs degrades rapidly during the early stage of F-T exposure, after which the degradation rate gradually slows, exhibiting a typical nonlinear attenuation pattern. Such a trend is consistent with the performance deterioration characteristics of most engineering materials subjected to long-term environmental actions, wherein damage accumulation is pronounced at the initial stage, followed by a gradual stabilization of degradation as the internal microstructure adjusts and damage zones become saturated.

### 5.4. Life Prediction

A F-T life prediction model [79] is employed, in which the stress ratio is integrally linked to the minimum ambient temperature and the maximum cooling rate under indoor and outdoor environmental conditions. This relationship enables the conversion of the number of F-T cycles occurring in natural environments into the equivalent number of F-T cycles obtained from standardized laboratory tests.

(16) $$k=\frac{\sigma_{max}}{\sigma_{i,max}}=\frac{{\left(Tln\frac{p_{\omega }}{p_{i}}\right)}_{in}}{{\left(Tln\frac{p_{\omega }}{p_{i}}\right)}_{out}}=\frac{{\left(\frac{d\theta }{dt}\right)}_{in}}{{\left(\frac{d\theta }{dt}\right)}_{out}}$$

where *σ*_max_ denotes the maximum compressive stress of the CG-IT SPBs under standardized indoor F-T conditions; *σ*_i,max_ is the maximum compressive stress of the CG-IT SPBs under a specific F-T regime under natural conditions; *T* is the minimum thermodynamic temperature under indoor or outdoor conditions (t + 273.15); *dθ*/*dt* is the maximum cooling rate under indoor or outdoor conditions (°C/min); and *p_ω_*/*p_i_* is the semi-empirical relationship proposed by E. W. Washburn, which describes the equilibrium between the freezing point of ice and the relative vapor pressure.

The relative mass loss data of CG-IT SPBs obtained from indoor F-T cycling tests were employed to establish a Wiener process-based degradation model. Furthermore, based on the average temperature data of six prefecture-level cities in the Inner Mongolia Autonomous Region from 2022 to 2025, a quantitative relationship between indoor experimental F-T cycles and outdoor natural F-T cycles was established, enabling the prediction of the remaining F-T service life of CG-IT SPBs under local climatic conditions in Inner Mongolia.

**Figure 16:**
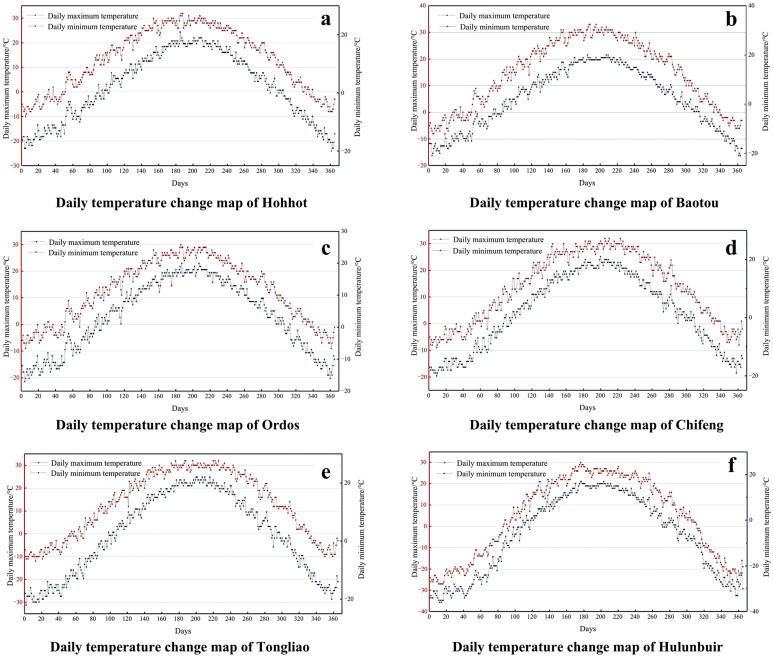
Variations in average temperature of six prefecture-level cities in the Inner Mongolia Autonomous Region from 2022 to 2025. (**a**) Hohhot; (**b**) Baotou; (**c**) Ordos; (**d**) Chifeng; (**e**) Tongliao; (**f**) Hulunbuir. Red dots represent daily maximum temperature, and blue dots represent daily minimum temperature.

In field conditions, temperature records are typically limited to daily maximum and minimum values. Therefore, the cooling rate of a single outdoor F-T cycle was approximated by the ratio of the daily temperature difference between the maximum and minimum temperatures to the corresponding time interval. Based on statistical data from the China Meteorological Administration, the average annual temperature variations of six prefecture-level cities in the Inner Mongolia Autonomous Region from 2022 to 2025 were compiled, as shown in Figure 16. As shown in the figure, time is referenced from January 1, and any day during which the minimum temperature falls below −5 °C while the maximum temperature exceeds 5 °C is regarded as a single actual outdoor F-T cycle [80].

Taking Baotou City in the Inner Mongolia Autonomous Region as an example, statistical data from the China Meteorological Administration indicate that the actual number of outdoor F-T cycles in Baotou in 2022–2025 was 23, with an average daily temperature difference of 13 °C/day. The maximum outdoor cooling rate can therefore be estimated as

(17) $${\left(\frac{d\theta }{dt}\right)}_{out}=\frac{13}{12\times 60}=0.018\;{}^{\circ}C/min$$

The indoor cooling rate is given by

(18) $${\left(\frac{d\theta }{dt}\right)}_{in}=\frac{32.5}{3\times 60}=0.181\;{}^{\circ}C/min$$

Through calculation, the actual number of F-T cycles, the average daily temperature difference, and the corresponding cooling rates for different prefecture-level cities in Inner Mongolia can be obtained (Table 2).

Thus,

(19) $$k=\frac{{\left(\frac{d\theta }{dt}\right)}_{in}}{{\left(\frac{d\theta }{dt}\right)}_{out}}=\frac{0.181}{0.018}\approx 10$$

Using Equation (16), the conversion ratios between indoor standard F-T cycles and outdoor natural F-T cycles for different prefecture-level cities were determined. That is, one F-T cycle under the standard experimental conditions employed in this study is equivalent to *k* F-T cycles under actual natural environmental conditions. According to the Wiener process-based analysis, when the failure criterion is taken as 0.4, the corresponding F-T cycle number is 10. This result suggests that one F-T cycle conducted under standard laboratory conditions is roughly equivalent to ten F-T cycles occurring in the natural environment of Baotou. On this basis, the frost-resistance service life of CG-IT SPBs under actual F-T conditions in Baotou can be determined using the following expression:

(20) $$t=\frac{kN}{n}$$

where *t* denotes the durability service life of the CG-IT SPBs under the actual F-T environment in Baotou (years); *n* is the number of F-T cycles experienced by the CG-IT SPBs under the real natural environment in Baotou (cycles); and *N* represents the number of F-T cycles at failure for the CG-IT SPBs under indoor F-T test conditions.

Through calculation, the remaining service life of CG-IT SPBs under the actual natural environments of different prefecture-level cities in the Inner Mongolia Autonomous Region can be obtained (Table 3).

In engineering applications, the selection of threshold values should consider the required safety margin and the local environmental conditions. According to the prediction results, for some cities, the theoretical service life at higher thresholds can reach several hundred years, which clearly deviates from the engineering expectations for conventional wall materials and represents the theoretical upper limit under idealized pure F-T conditions. Such values should not be directly used as a basis for practical design. In contrast, the predicted service life for Baotou and Chifeng under moderate threshold values is approximately 34 and 42 years, respectively, which is broadly consistent with the empirical service life of ordinary wall materials in harsh F-T environments of cold regions. Nevertheless, these absolute values still need to be further adjusted by considering multi-factor coupling effects, such as salt erosion and wet–dry cycling.

It should be noted that the F-T lifetime prediction model developed in this study considers only F-T cycles as a single environmental factor. In practical engineering applications, particularly in cold regions such as Inner Mongolia, wall materials are often subjected to the coupled effects of F-T cycles and salt erosion, where the interaction between soluble salts and ice crystal growth is especially critical.

When soluble salts are present in the pores of bricks, the salt solution lowers the freezing point of water, thereby altering the effective temperature range of F-T cycles. More importantly, during cooling, the salt solution may crystallize or become supersaturated, and the crystallization pressure generated by salts combines with the expansion pressure of ice crystals. This synergistic effect acts on the pore walls, promoting the initiation and propagation of microcracks. Consequently, the damage rate under a coupled salt–freeze environment is generally much higher than that under pure F-T conditions [81,82,83]. In addition, salt erosion can accelerate surface spalling and strength loss, and its damage patterns bear some resemblance to the F-T damage observed in this study.

Other factors, such as ultraviolet radiation, wet–dry cycling, and microbial activity, may also indirectly influence brick durability, but their coupling mechanisms are more complex and are not addressed in detail in this work. Overall, the model predictions in this study should be regarded as theoretical reference values under pure F-T conditions. Future research should design accelerated tests coupling F-T cycles with salt immersion to systematically investigate the synergistic mechanisms of salt and ice, and to develop a multi-factor coupled lifetime prediction model.

## 6. Conclusions

This study combined systematic experiments with theoretical analysis. It focused on the preparation method, F-T damage evolution, and frost-resistance life prediction of CG-IT SPBs. The main conclusions are listed below.

(1) By using CG, ITs, and shale as raw materials, porous bricks that meet relevant standards can be produced. This result is achieved through proper control of mix proportions and sintering conditions. The sintering temperature determines the initial pore structure and glass phase content of the CG-IT SPBs, and it is conducive to the development of microstructural features that enhance frost resistance and durability. Under unfrozen conditions, the bricks show good physical and mechanical properties. This behavior indicates that CG, ITs, and shale have chemical complementarity. Together, they can replace traditional clay-based materials. This result confirms the feasibility of high value-added use of bulk industrial solid wastes in building materials.
(2) Under F-T cycling, damage in CG-IT SPBs develops gradually from the surface to the interior. At the macro level, this damage appears as mass loss, reduced compressive strength, and higher water absorption. At the micro level, it is linked to the breakdown of the glassy phase and the formation and connection of pores. Among all measured indicators, compressive strength is the most sensitive to F-T action. Its damage degree is much higher than that of other parameters. This result suggests that F-T action mainly weakens the mechanical integrity of the material. In contrast, bulk density changes only slightly. This result means that the overall shape of the specimens stays relatively stable during the early F-T stage.
(3) The Wiener process-based F-T damage model effectively captures the stochastic behavior and cumulative characteristics of performance deterioration. By fitting data from accelerated F-T tests, the reliability function and probability density function were obtained. These functions were then combined with local climate conditions to estimate the theoretical service life under natural F-T environments. This life prediction method provides a new way to evaluate durability and guide the design of similar solid waste-based building materials.

This study has achieved certain progress in the preparation, F-T damage evolution, and service life prediction of CG-IT SPBs, yet several issues remain to be further explored. Future research will focus on the following aspects:

(1) Experiments combining thermogravimetric analysis (TGA) and differential scanning calorimetry (DSC) will be conducted to elucidate the effects of sintering temperature on microstructure and macroscopic properties, thereby determining the optimal sintering temperature range. In parallel, the volumetric shrinkage of bricks under different processing conditions will be systematically measured, establishing correlations between sintering shrinkage, pore structure, and F-T durability.
(2) X-ray diffraction (XRD) coupled with Rietveld full-profile refinement will be employed to quantify the crystalline phase composition and amorphous glass content of the raw materials and sintered bricks. This will clarify the thermodynamic stability of mineral phases during sintering and their mechanisms of resistance to F-T damage.
(3) Experiments will investigate the synergistic damage mechanisms induced by soluble salts and ice crystallization. A multi-factor coupled service life prediction model considering salt erosion and wet–dry cycling will be established, providing predictions that more closely reflect actual service conditions.

Through these studies, the preparation process of CG-IT SPBs will be further optimized, the theoretical framework for F-T damage mechanisms will be refined, and more comprehensive scientific guidance will be provided for their high-value engineering applications in cold regions.

## Figures and Tables

**Table 1:** Chemical composition of raw materials.

Raw Material	Content of Chemical Components (%)									
	SiO_2_	Al_2_O_3_	Fe_2_O_3_	CaO	MgO	K_2_O	Na_2_O	SO_3_	TiO_2_	LOI
Coal gangue	48.94	15.22	6.24	1.42	1.68	2.65	1.32	0.05	0.83	21.50
Iron tailings	57.22	19.06	11.81	2.25	1.14	2.50	0.50	0.01	−	4.80
Shale	58.35	13.42	5.33	1.68	1.37	2.78	1.76	0.57	1.08	13.20

**Table 2:** Actual F-T cycles, average daily temperature differences, and cooling rates of different prefecture-level cities in Inner Mongolia.

Region	The Actual Number of Freeze–Thaw Cycles/Times	Average Daily Temperature Difference °C/Day	The Cooling Rate is °C/h.
Hohhot	12	10.92	0.91
Bao Tou	23	13	1.08
Ordos	5	11.4	0.95
Chifeng	20	12	1
Tongliao	9	11.56	0.96
Hulunbeier	9	12.78	1.07

**Table 3:** Remaining service life of CG-IT SPBs under the actual natural environments of different prefecture-level cities in Inner Mongolia.

Region	*K*	*F_k_* = 0.2 (Years)	*F_k_* = 0.3 (Years)	*F_k_* = 0.4 (Years)
Hohhot	11.9	24.2	77.1	186.8
Bao Tou	10.0	10.6	33.8	81.9
Ordos	11.4	55.6	177.4	429.6
Chifeng	10.8	13.2	42.0	101.7
Tongliao	11.3	30.6	97.7	236.5
Hulunbeier	10.1	27.4	87.3	211.4

## Data Availability

The raw data supporting the conclusions of this article will be made available by the authors on request.
